# Chorion-Epithelioma, Vesicular Mole, and Lutein Cysts

**Published:** 1904-12-24

**Authors:** 


### CHORION-EPITHELIOMA, VESICULAR MOLE, AND LUTEIN CYSTS.

Although happily not a frequent malady, chorion-epithelioma is one of the most malignant tumours from which human beings are liable to suffer. Any suggestion which holds a promise of more successful therapeutic measures in the future will therefore be worthy of meditation. In the current number of the *Practitioner* Dr. Cuthbert Lockyer calls attention to two points in the pathology of the condition which may have an important bearing on future practice. In the first place he refers to the relationship between vesicular mole and chorion-epithelioma, and secondly he describes his own observations upon the co-existence of lutein cysts or of excess of lutein tissue in the ovaries with chorion-epithelioma. Marchand was the first to show that in vesicular mole the all-important change is an abnormal proliferation of the chorionic epithelium. Then followed the discovery of a close resemblance between the proliferating epithelium of a vesicular mole and the cellular structures of chorion-epithelioma; in fact the latter appeared to be merely an exaggeration of the former, the difference being only one of degree. Further, histological evidence was forthcoming of invasion of the wall of the uterus by the proliferating cells of a vesicular mole, a discovery which "gave a new clinical significance to the nature of vesicular disease of the chorion; it showed that, if proliferation of the epithelium of the villi be carried far enough, it gave rise to one of the most malignant of all known tumours; and that, given a hydatid mole, it was only a question of degree whether the disease was confined to the placenta or spread to the uterus and became malignant." According to this view a vesicular mole may be regarded as the incipient stage of chorion-epithelioma.

What is the cause of the disease of the ovum? This question leads up to the second part of Dr. Lockyer's paper, in which he deals with the associated over-development of lutein tissue in the ovaries. In a number of instances, both of vesicular mole and chorion-epithelioma, cystic disease of the ovaries has been noted. Dr. Lockyer has examined some of these cystic ovaries, and has also cut sections of ovaries removed from cases of vesicular mole and chorion-epithelioma where no cysts were present, and his observations, so far as they have gone, afford support to the proposition that in all cases of vesicular mole the ovaries contain an excess of lutein tissue. And it can hardly be doubted that the ovarian changes precede those in the uterus, for there are cases on record where the presence of ovarian swellings had been detected as long as a year before the signs of pregnancy and vesicular disease became apparent. On the whole, Dr. Lockyer's paper points to the need for an extensive and critical inquiry into the pathology of vesicular mole and chorion-epithelioma, for the possibility of the one being a "precancerous" stage of the other is probable, and if this supposition be confirmed, the removal of the uterus and adnexa in cases of vesicular mole, would seem likely to offer a chance of saving the patients from subsequent chorion-epithelioma.

